# Implementing a successful proactive telephone breastfeeding peer support intervention: volunteer recruitment, training, and intervention delivery in the RUBY randomised controlled trial

**DOI:** 10.1186/s13006-021-00434-9

**Published:** 2021-12-01

**Authors:** Heather A. Grimes, Helen L. McLachlan, Della A. Forster, Fiona McLardie-Hore, Kate Mortensen, Touran Shafiei

**Affiliations:** 1 Judith Lumley Centre, La Trobe University, Bundoora, Victoria Australia; 2 School of Nursing & Midwifery, College of Science, Health and Engineering, La Trobe University, Bundoora, Victoria Australia; 3 La Trobe Rural Health School, PO Box 199, Bendigo, Victoria 3550 Australia; 4 The Royal Women’s Hospital, Grattan St and Flemington Roads, Parkville, Victoria Australia; 5 Australian Breastfeeding Association, Melbourne, Australia

**Keywords:** Peer support, Telephone support, Breastfeeding, Implementation

## Abstract

**Background:**

The RUBY randomised controlled trial demonstrated the benefit of proactive telephone peer support in promoting breastfeeding continuation in a setting with high breastfeeding initiation, where typically this is difficult to achieve. This paper describes the implementation and delivery of the peer support intervention with a focus on recruitment, training, and support of peer volunteers, and includes a description of the key components of the calls.

**Methods:**

Data collection occurred between December 2012 and June 2016 in Melbourne, Australia. Volunteers completed enrolment forms at the training session and recorded data related to each call in a Call Log maintained for each mother supported. Data were summarised using descriptive statistics and responses to open-ended questions analysed using content analysis.

**Results:**

A total of 693 women expressed interest in the peer support role, with 246 completing training, that is, 95% of whom supported at least one mother. Each supported a mean of two mothers (range 1 to 11). Training session topics included respecting individual values, using positive language, confidence building, active listening, empathetic support, and normal baby behaviour. There were 518 periods of support where at least one call was made between a volunteer and a mother to whom she was allocated. Of the 518 periods of support, 359 Call Logs (69%) were returned. The 359 call logs recorded a total of 2398 calls between peers and mothers. Call length median duration was 12 min (range 1 to 111 min). Volunteers perceived the most valued aspects of the calls were the provsion of ‘general emotional support’ (51%) and ‘general information/discussion about breastfeeding’ (44%). During the first call, mothers raised questions about ‘nipple pain/ damage’ (24%) and 'general breastfeeding information’ (23%). At ≥12 weeks postpartum, issues raised related to ‘normal infant behaviour’ (22%), ‘feed frequency’ (16%), and ‘general breastfeeding information’ (15%). Volunteers referred women to other resources during 28% of calls, most commonly to the Australian Breastfeeding Association.

**Conclusions:**

Our findings demonstrate that the RUBY trial was feasible and sustainable in terms of recruiting volunteers who were willing to participate in training and who proceeded to provide peer support. Call content was responsive to the evolving breastfeeding information needs of mothers and the provision of emotional support was perceived by volunteers to be important.

**Trial registration:**

Australian New Zealand Clinical Trials Registry, ACTRN 12612001024831.

## Background

The Ringing up About Breastfeeding Early (RUBY) randomised controlled trial (RCT) conducted in Melbourne, Australia, demonstrated that in the context of a high rate of breastfeeding initiation, proactive telephone peer support provided by a peer volunteer in the first six months postpartum was an effective intervention for increasing breastfeeding maintenance [1]. In the RUBY study, significantly more infants of women assigned to proactive telephone peer support were receiving *any* breast milk at six months of age compared to women assigned to usual care [1]. High level quantitative outcomes such as those reported in the RUBY RCT make a contribution to the evidence for breastfeeding peer support interventions [2–4], and it is crucial to understand how interventions shown to improve outcomes were implemented, to ensure they can be replicated and sustained.

Lack of detail when reporting processes and monitoring fidelity of interventions influences interpretation of study findings [5]. This issue is particularly relevant when designing interventions that have wide heterogeneity, as is the case for peer support RCTs [4] and for RCTs that lie at the pragmatic end of the pragmatic- explanatory spectrum [6]. In relation to studies of breastfeeding peer support, there has been a call to provide details about delivery of the support, including who delivered it, how it was delivered, the intensity, and whether it was proactive or reactive [7] [4]. For example, despite ‘experiential knowledge’ being central to the concept of peer support [8], the personal infant feeding experience of peers is only occasionally reported [9, 10], and the length of breastfeeding experience is frequently unspecified [11]. Thompson and Trickey [7] highlight the limitations in focusing only on outcomes from experimental breastfeeding peer support studies, without considering contextual factors and key points of variation between studies such as the characteristics and training of peers. Omission of details regarding intervention delivery has also been identified as a limitation when systematically reviewing evidence from peer support RCTs [3, 4].

The aim of this paper is to describe factors related to the implementation of the RUBY peer support intervention [12]. The four key components reported here are: i. key aspects of recruitment, training and support of the peer volunteers; ii. details regarding the key topic areas discussed during the calls as well as referrals suggested by volunteers; iii. Volunteers’ perceptions of the value of the calls to mothers; and iv. details regarding the role of the peer volunteer coordinator. The views and experiences of the peers have been reported in separate publications [13, 14].

## Methods

### Study context - RUBY study overview

The detailed study protocol for the RUBY randomised controlled trial (RCT) is published elsewhere [12]. Briefly, RUBY was a two-arm RCT of a proactive telephone breastfeeding peer support intervention for women who were recruited from the postnatal units of three public hospitals in the state of Victoria, Australia (*n* = 1152). Women were eligible for inclusion if they were first time mothers, admitted as public patients to the postnatal units of the participating hospitals, were proficient in English and were intending to breastfeed. Women were randomly allocated to receive either usual care (*n* = 578) or the peer support intervention (*n* = 574). In this setting, ‘usual care’ comprised a hospital stay of up to 48 hours following vaginal birth and 72 hours following caesarean section. Following discharge, women could access hospital-based breastfeeding services including lactation consultants. Peer support was provided by volunteer women recruited from the community. Volunteers were guided by the RUBY call schedule. The volunteer made the first contact within four to six days of birth and followed up with a second call within three to four days of the first. Calls were then weekly for 12 weeks and then three to four weekly until the baby was six months of age. They were advised that the actual call frequency could also be responsive to the mothers’ needs [12].

#### The peer volunteers

Women were eligible to be peer volunteers if they had breastfed a baby for at least six months, were keen to support other mothers, and were not breastfeeding ‘experts’ or ‘counsellors’ [12]. In the early weeks of volunteer recruitment, several health professionals, including midwives, student midwives, nurses and general practitioners expressed interest in the peer support role. It was difficult to quantify the amount of breastfeeding education they had received in their professional roles, therefore, to ensure the RUBY peers possessed mainly experiential knowledge, health professionals or breastfeeding counsellors who had received more than eight hours of breastfeeding training were considered ineligible. After initial screening, volunteers were provided with an overview of the program requirements and invited to attend a RUBY volunteer training session. Further screening of volunteers was undertaken at the training session and focused on observing communication skills and English proficiency. These were considered core skills given volunteers would be delivering proactive telephone support. Further details of the training and support provided to peer volunteers is detailed in the Results.

### Data collection

Data related to the volunteers were collected from the time of their initial expression of interest in the role. At this point, their name and contact details were recorded in an Access database and each was ascribed a unique study number. Following screening by the volunteer coordinator, further demographic details and responses to eligibility criteria screening questions were recorded. At the conclusion of the training session, those volunteers who wished to pursue the role were asked to complete a volunteer enrolment form, and a privacy and confidentiality consent form. Following the training session, the volunteer coordinator entered all volunteer data into the database.

Data related to each call were recorded by the volunteer in pre-coded Call Logs developed for the RUBY trial and maintained by volunteers for each woman supported. Hard copies of the Call Logs were provided to the volunteers. They could request an electronic version if preferred. Each Call Log included the date, time and duration of each call, who initiated the call, whether the volunteer felt the mother valued the call and the reason for this response. Topics discussed during the call, referrals to other services and information given to the women, including recommended fact sheets and websites were recorded.

All Call Logs were assigned a unique numerical identifier when the period of support commenced. When each Call Log was returned, all data were entered into a password secured Access database [15], identifiable only by the pre-assigned number. Email reminders were sent to volunteers who had not returned their Call Logs to encourage them to do so. If a Call Log was not returned, this was noted in the database. If a volunteer was not able to establish contact with a mother, this was also recorded in the Call Log database.

### Data analysis

Quantitative data were analysed using Stata Version 15 [16]. Frequencies, percentages, and means were used to describe the data. Responses to open-ended questions were analysed using simple content analysis [17].

### Ethics

Research ethics approval was obtained from La Trobe University (12–08), Royal Women’s Hospital (12/25), Western Health (HREC/12.WH/107) and Monash Health (12251B). The RUBY trial was registered with the Australian and New Zealand Clinical Trials Registry prior to commencement (ACTRN12612001024831).

## Results

### Peer recruitment and training

**Fig. 1:**
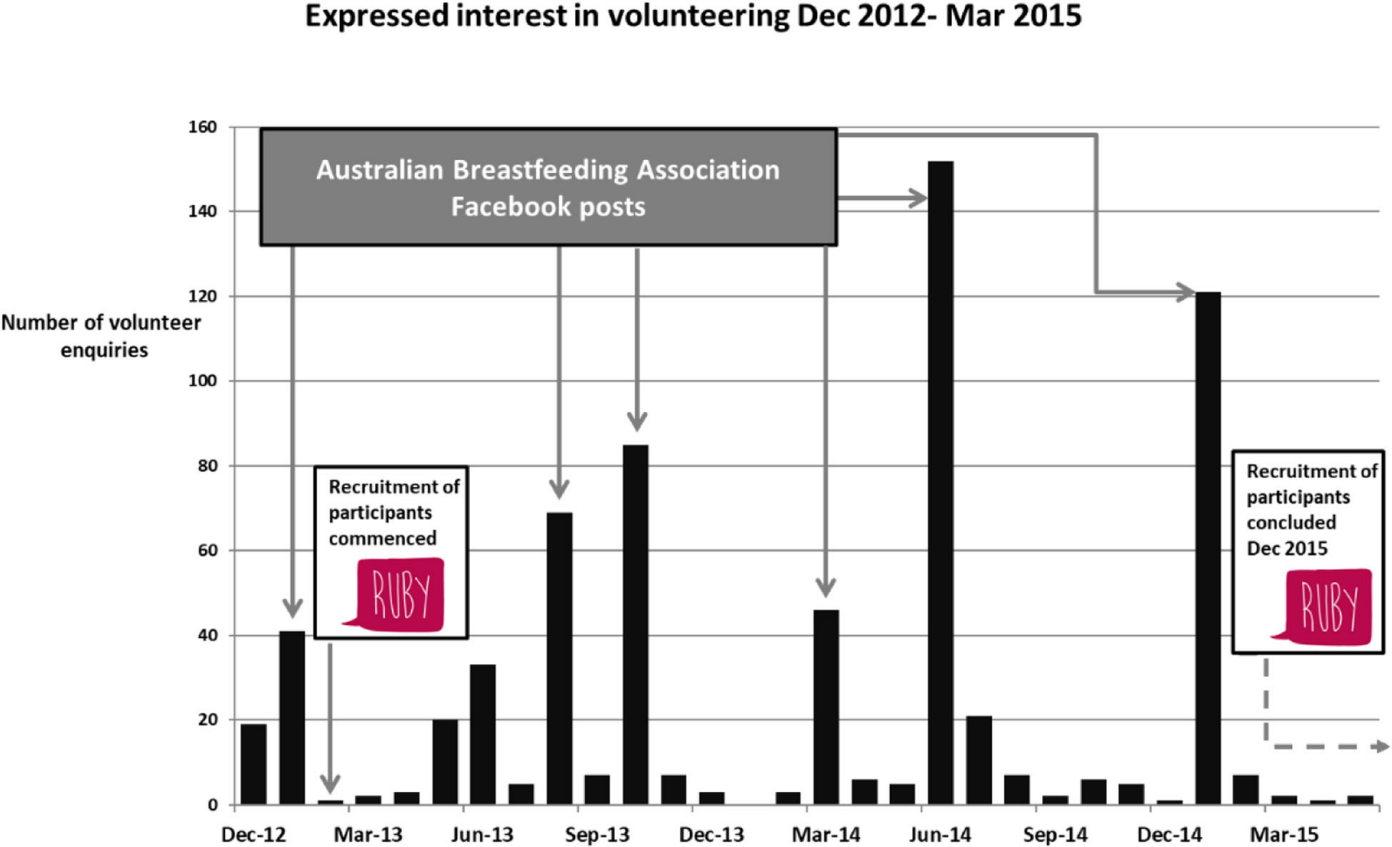
Volunteer enquiries in relation to ABA Facebook post

The first volunteers were recruited on 21 December 2012, with the first training session date on 16 January 2013. Recruitment of participants commenced on 14 February 2013 and concluded on 15 December 2015. Recruitment of volunteers commenced with dissemination of hardcopy flyers advertising the study to Maternal and Child Health (MCH) centres. As the study progressed, this method was replaced with electronic flyers posted to Australian Breastfeeding Association (ABA) online platforms. The ABA is a non-profit, volunteer organisation and Australia’s largest breastfeeding information and support service. This was a successful strategy and each post resulted in a surge of interest from potential volunteers (Fig. 1). Volunteers either emailed or expressed interest in the role by contacting the volunteer coordinator by phone. They were screened for eligibility and their contact details recorded. Eligible women were then invited to attend a training session.

**Fig. 2:**
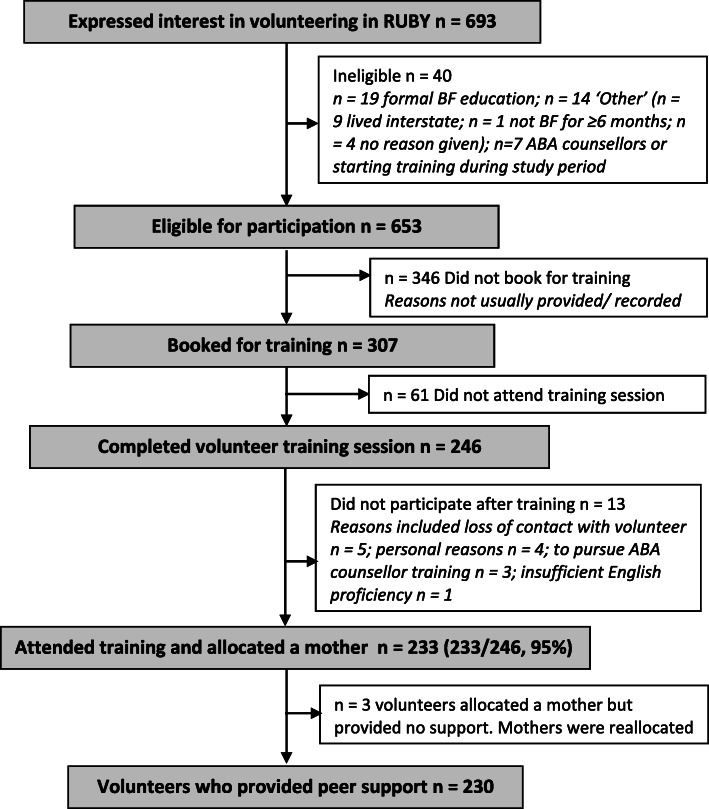
Recruitment and training of volunteers

Over the course of the study, a total of 693 women expressed interest in volunteering for the RUBY study, and of the 307 (44%) who booked into a training session, 246 (80%) attended (Fig. 2). Of these, most volunteers (*n* = 233, 95%) were allocated a mother. We do not have complete data regarding reasons why women who expressed interest not taking the next step and booking into a training session as we often had no further contact beyond their initial expression of interest. For those for whom a reason was known, the most commonly cited reasons were illness or changed work commitments.

#### The training session

Peers attended a four-hour training session facilitated by an educator from the ABA and attended by one of the RUBY chief investigators, the project coordinator, and the peer volunteer coordinator. The training session was based on an existing ABA program and took place at a centrally located venue, close to public transport and with convenient parking. Overall, 24 training sessions were conducted between January 2013 and May 2015, approximately one every four to 8 weeks.

Each session commenced with a discussion of the volunteers’ personal experiences of breastfeeding and their motivation for participating in the study. The chief investigator presented the rationale and aims of the study, the sites involved, and a brief overview of what volunteering would entail including anticipated time commitment. The volunteer coordinator outlined the process of allocating mothers to volunteers and the support volunteers would receive during participation. At the end of the session, participants who were interested in being peer supporters completed an enrolment form and signed a Privacy and Confidentiality agreement.

Training session topics included respecting the beliefs and values of others, using positive language, confidence building, active listening and empathy, encouraging and supporting new mothers, and normal baby behaviour. Activities such as showing participants a series of photographs depicting various infant feeding scenarios were used to stimulate discussion of values and norms. The aim was to highlight pre-existing attitudes to feeding choices and to clarify personal values and judgements. The power of positive language to teach, build confidence and re-frame challenges was demonstrated and discussed using examples. Role-play scenarios were used to practice active listening and providing empathic responses. Information was presented about expected baby behaviour in relation to breastfeeding such as frequency of feeds, overcoming nipple pain, hunger cues, signs of adequate nutritional intake, reasons a baby might cry, and strategies for soothing a fussy baby. Problem solving and recognising the need for referral were explored, and links to resources providing quality breastfeeding information provided.

The procedure for how volunteers were allocated mothers for peer support has been described elsewhere [13]. Briefly, the volunteer coordinator received the new mother’s contact details following recruitment and randomisation to the intervention group. The mother was allocated to the ‘next available’ volunteer. Each mother was allocated one volunteer, and it was expected that the relationship would continue for the duration of the six-month period of support.

#### The RUBY volunteer handbook

A 32-page *RUBY Volunteer Mother’s Information Manual* was developed collaboratively by the RUBY study team and the Australian Breastfeeding Association. It was also informed by the *Mother Helping Mothers with Postpartum Depression* peer volunteer training manual developed by Professor Cindy-Lee Dennis (Dennis, C.L., personal communication to Professor Della Forster - 18 December 2012). Volunteers received a printed copy of the manual when they attended the training session. The manual reiterated key messages from the training session and listed appropriate sources of support to which they could refer mothers e.g. useful web pages and organisations such the ABA and Maternal and Child Health services (Table 1).

**Table 1:** Topics in the RUBY volunteer training manual

Section i	About the study
	• Which organisations are involved in the study?
	• How many women will be involved?
Section ii	Being a RUBY volunteer mother
	• What will be expected of me?
	• Who can be a volunteer?
	• What is the role of the volunteer coordinator?
	• Who will I contact if I no longer want to be involved in the study?
	• What do I do when the period of support ends?
	• Who will support me?
Section iii	Getting connected – staying connected: developing a relationship with the new mother
	• Getting connected
	• Staying connected
	• How much time will it take to “stay connected”?
	• Developing a relationship with the mother
Section iv	Skills and techniques to effective telephone support
	• Learning about respecting other people’s values and cultural beliefs
	• Language – what are we really saying
	• Building confidence
	• Listening
	• Empathy is showing a mother you understand
	• Babies – what is normal
	• Breastfeeding and work
	• Practising being a volunteer breastfeeding supporter – role plays
Section v	Resources and support services

#### Training and support provided to the volunteers

Table 2 provides a summary of the support provided to volunteers. A volunteer coordinator (HG) who was an experienced midwife was appointed for the duration of the RUBY trial. The role included screening prospective volunteers, participating in training sessions, facilitating contact between peers and mothers, and responding to concerns raised by volunteers. The volunteer coordinator maintained regular contact with volunteers, commencing approximately seven days after allocation, to confirm contact with the mother had been made. The volunteer coordinator followed up with weekly and then monthly contacts either by phone or email and could be contacted by phone or email as required. In addition, the volunteer coordinator coordinated twice yearly ‘social’ events for volunteers to foster collaboration and support between volunteers.

**Table 2:** Summary of training and support provided to the volunteers

Component	Description	When provided	Volunteers involved
**Face to face training sessions**	4-h training session facilitated by research team and ABA facilitator	Prior to commencing peer support role	Mandatory for all volunteers
**Training manual**	A hardcopy of the 32-page RUBY Volunteer Mother’s Information Manual	Given to all volunteers during the training session	All eligible volunteers attending the training session
**Volunteer social events**	Informal morning-tea facilitated by volunteer coordinator and chief investigator	Approximately every six months for duration of study	Optional invitation to all volunteers providing peer support
**Regular phone/email contact from volunteer coordinator**	Phone or email contact with volunteers by volunteer coordinator during periods of support.	Within one week of allocation of a mother. Another call made a week later and then monthly contact during period of support.	All volunteers actively providing peer support
**Financial reimbursement**	$50 AUD reimbursement for calls made during each period of support	At the conclusion of each period of support	All volunteers actively providing peer support could submit forms for reimbursement

When any period of support finished, the volunteer was sent a pack containing forms to claim reimbursement for calls, a new Call Log if the volunteer was available for allocation of mothers in the future, and a postage paid envelope for return of the completed Call Log. A thank you letter was included, encouraging volunteers to return the Call Logs even if the period of support was brief. Volunteers were offered $50 AUD reimbursement for each completed period of support, subject to return of Call Logs.

#### Demographic characteristics of participants

The demographic characteristics of all volunteers who supported at least one mother in the RUBY study are presented in Table 3. The mean age of volunteers was 33.9 years (standard deviation (SD) 5.0 years), and 82% were born in Australia (189/230). The majority had one child (52%) and the mean age of their youngest child at enrolment was 16.8 months. We asked volunteers to tell us the length of their longest experience of breastfeeding an individual child. The mean was 15.7 months and ranged between 6 and 60 months. A little over one third of volunteers were members of the ABA at the time of enrolment (120/230).

**Table 3:** Characteristics of peer supporters who supported at least one mother in the RUBY RCT^a^

Participant characteristic	n (*n* = 230)	*%*
Peer supporter’s age in years (mean = 33.9)		
18–25 years	3	*1.3*
26–34 years	125	*54.4*
≥ 35 years	102	*44.4*
Number of children at time of enrolment (range 1–7)		
One child	120	*52.2*
Two children	85	*37.2*
More than two children	25	*10.9*
Number of children breastfed (, range 1–7)		
One child	127	*55.2*
Two children	79	*34.3*
More than two children	25	*10.9*
Youngest baby’s age at time of enrolment (months) (range 3–312)	mean 16.7, SD 26.8	
Longest duration of breastfeeding an individual child (months) (range 6–60)	mean 15.7, SD 7.5	
Country of birth		
Australia	189	*82.2*
Other (UK = 11; NZ = 5; USA = 3; India = 2; Lebanon = 2; Argentina, Afghanistan, Belarus, Brazil, China, Fiji, Germany, Ireland, Singapore, South Africa, South Korea and Switzerland all = 1; Not stated = 6)	41	*17.8*
Current member of Australian Breastfeeding Association	80	*34.8*
Total number of mothers each peer supported in RUBY RCT (range 1–11)	mean 2.5, SD 1.7,	

^a^Ringing up About Breastfeeding Early randomised controlled trial

### Intervention delivery

In this section we present findings related to delivery of the intervention as reported by volunteers in Call Logs. There were 574 new mothers allocated to the peer support intervention group in the RUBY study and 579 ‘periods of support’ (five mothers were allocated to a second volunteer when the first couldn’t continue providing peer support). Calls for each ‘period of support’ were recorded in a Call Log assigned a unique identifier. Of the 579 periods of support, in 61 cases (11%), contact was never established [1], leaving 518 periods of support in which at least one call was made. In total, 359/ 518 (69%) Call Logs were received from volunteers who had made at least one call. The $50 reimbursement offered for each mother supported was claimed by volunteers for 222/518 (43%) periods of support.

Overall, 2398 calls were recorded in the Call Logs, ranging in duration from 1 to 111 min, with a median duration of 12 min. The data related to missed calls and text messages were inconsistently reported and is not reported here. For example, some volunteers recorded each call attempt while others only recorded the first attempt but noted comments like ‘tried lots of times’. The section for recording text messages was added several months into the trial following feedback from volunteers and is therefore incomplete. The texts were also recorded inconsistently, again with some volunteers recording each text, and others noting only the first text. After each call, volunteers were asked to record in their Call Log if they thought the woman valued/ appreciated the call. If they answered yes (*n* = 2300), a closed-ended question explored which aspect of the call the mother valued (Table 4). From the volunteer’s perspective, women appreciated the ‘general emotional support’ (51%) and ‘general information/discussion provided about breastfeeding’ (43.6%). Being ‘someone to talk to but not necessarily about breastfeeding’ also seemed to be valued by recipients (42%).

**Table 4:** Aspects of the call the woman valued/appreciated (as assessed by peer volunteer)

Aspect valued in call	n (*n* = 2320)	%^a^
General emotional support	1182	51.0
General information/discussion about breastfeeding	1011	43.6
Someone to talk to but not necessarily about breastfeeding	975	42.0
Responses to specific breastfeeding related questions/ concerns raised by the woman	614	26.5
Unsure	54	2.3
Other	161	6.9

^a^More than one response could be selected, so % may add to more than 100

#### Intervention delivery: content of calls

Volunteers were asked to indicate the main concerns raised by the woman during each call, and were provided with a pre-coded list of topics (as well as having an option of ‘other’). Of the 2398 calls, there were a total of 1576 (66%) calls during which a woman raised a specific topic (Table 5). Of those who raised concerns, we examined these responses across ‘all calls’ as well as by looking at topics raised ‘during the first call’ (*n* = 359), ‘during calls when baby age was less than 12 weeks of age (excluding the first call)' (n = 1459) and finally, during calls where ‘baby age was equal to or greater than 12 weeks’ (*n* = 570). The rationale for categorising responses into these timepoints was to explore if there was any change in topics over time.

**Table 5:** Main issues raised by women during calls (as per provided checklist)

5.Topic raised	During first call (***n*** = 359)		Baby age < 12 weeks (excluding first call) (***n*** = 1459)		Baby age ≥ 12 weeks (***n*** = 570)		All calls (***n*** = 2398)	
	n	*%*^*a*^	n	*%*^*a*^	n	*%*^*a*^	n	*%*^*a*^
Number of calls during which at least one specific concern was raised by mother	272	*77%*	977	*67*	321	*56*	1576	*66*
**Specific concern raised**								
Nipple pain/ damage	87	*24*	128	*9*	20	*6*	235	*10*
Feed frequency	80	*22*	228	*16*	68	*12*	376	*16*
Positioning/attachment	78	*22*	107	*7*	4	*< 1*	189	*8*
General BF information	83	*23*	218	*15*	65	*11*	366	*15*
Normal infant behaviour	71	*20*	341	*23*	106	*19*	518	*22*
Supply & demand	66	*18*	179	*12*	38	*6*	283	*12*
Expressing	63	*18*	189	*13*	48	*8*	300	*13*
Not enough milk	54	*15*	138	*9*	35	*6*	227	*9*
General concern/ anxiety	34	*9*	86	*6*	21	*6*	141	*6*
Engorgement	31	*9*	64	*4*	6	*1*	101	*4*
Mother’s health problem	25	*7*	57	*4*	20	*6*	*102*	*4*
Nipple shield	23	*6*	44	*3*	2	*< 1*	69	*3*
Oversupply	18	*5*	53	*4*	7	*1*	78	*3*
Baby unwell	14	*4*	60	*4*	17	*5*	91	*4*
Tongue-tie	12	*3*	30	*2*	2	*< 1*	44	*2*
Bottle/ formula feeding	7	*2*	44	*3*	20	*6*	71	*3*
Mastitis	5	*1*	41	*3*	7	*1*	53	*2*
Nipple/ breast thrush	5	*1*	32	*2*	9	*3*	46	*2*
Infant wellbeing	3	*< 1*	25	*2*	11	*3*	39	*2*
BF in public/ travelling	1	*< 1*	20	*1*	4	*1*	25	*1*
Return to work	0	*0*	20	*1*	12	*4*	32	*1*
Introducing solids/ weaning	0	*0*	6	*< 1*	49	*9*	55	*2*
Other	1	*< 1*	10	*< 1*	7	*1*	18	*< 1*

^a^Respondents could tick more than one option so % could add to more than 100

Overall, ‘normal infant behaviour’ (22%), ‘feed frequency’ (16%), and ‘general breastfeeding information’ (15%) were the most frequent topics discussed. These continued to be the most frequent topics discussed at each time-point except during the first call, when ‘nipple pain/ damage’ (24%) was most frequently discussed (Table 5). ‘Other’ topics not in the pre-coded list were mostly related to bottle/ formula feeding, introducing solids/ weaning, infant well-being, return to work and breastfeeding in public.

#### Referrals

If a woman raised a concern that was beyond a volunteer’s experience or was an issue better addressed by a professional or expert, the volunteer referred the woman to health or support services based on a list of recommended services. Volunteers reported referring women to one or more services during 673 of the 2398 calls recorded in the Call Logs (Table 6). The most common referral was to the ABA (56%). Other referrals were made to the Maternal and Child Health service, general practitioners, and lactation consultants.

**Table 6:** Referrals made by volunteers during calls to mothers

Referral organisation/person/information source	n (*n* = 673)	%^a^
Australian Breastfeeding Association	378	56
Maternal and Child Health service	254	38
General Practitioner	116	17
Hospital lactation service	57	9
Private lactation consultant	51	8
Hospital service e.g. emergency department	11	2
Other ^**b**^	133	20

^a^Respondents could tick more than one option so % could add to more than 100

^**b**^Includes referrals to specific websites (*n* = 61), neonatal sleep related resources (*n* = 17), pharmacists and hospital drug information call-lines (*n* = 15), health professionals such as paediatricians (*n* = 14), local government resources such as maternal and child health clinics or breastfeeding drop in centres and mothers’ groups (*n* = 11), books (*n* = 4) and various other resources such as ‘google’ and ‘baby wearing’ products (*n* = 16)

## Discussion

This paper describes key components involved in implementing the proactive telephone peer support intervention delivered in the RUBY RCT. In this paper we have focused on processes related to the peer volunteers, including their recruitment, training and support, and the role of the volunteer coordinator. These findings address a call for more detail on implementation of peer support interventions, which has been identified as a limitation when reviewing evidence from peer support RCTs [3] . Overall, we found that interest in participating in the peer support program within the RUBY study was strong and once women completed the training session, they were likely to provide support.

From the outset, our collaborative research partnership with the Australian Breastfeeding Association (ABA) provided multiple practical benefits when recruiting volunteers and developing the training session and manual. The important benefits obtained through engagement with existing local services and infrastructure with compatible aims has been described in previous peer support research [2, 13, 18]. The ABA is a national not-for-profit organisation providing community-based support for breastfeeding women [19]. The reach of the ABA online platforms provided significant leverage when recruiting peer supporters and using existing ABA resources, supported development of the RUBY training session and manual.

Following recruitment of peers, a second crucial step in breastfeeding peer support programs is linking peers with new mothers. How this is achieved depends on the design of the program, but all programs offering one to one peer support need a clear strategy for ensuring peers are aware of breastfeeding mothers, and provided with a means of contacting them [20]. As identified by Trickey et al., [21], delays in referring women caused by poor referral pathways may delay support during the early postnatal period when women are most vulnerable to stopping breastfeeding [22]. Within the bounds of the RUBY study, this was achieved by research midwives recruiting mothers in the postnatal units of participating hospitals and the supporting role of the volunteer coordinator. Scale up of a similar program would need to consider how the link between mothers and peers would be facilitated.

The aim of the RUBY training session was to ensure peers could provide a supportive environment for new mothers to address the complexities of breastfeeding within their own unique contexts, while providing experiential insights that would assist this process. Having practical experience of a phenomenon does not necessarily equate to having the ability to share this experiential knowledge effectively [23, 24]. A key function of the RUBY training sessions was to explore the volunteers’ attitudes (recognising own attitudes to infant feeding), skills (active listening, re-appraisal of concerns), and knowledge (common breastfeeding issues attitudes) in relation to breastfeeding. To some extent, the group training sessions provided the opportunity for peers to develop collective knowledge by hearing the stories of other peers. Sharing breastfeeding stories within a group may enable individuals to exceed the boundaries of their personal experience through the development of collective experiential knowledge [24].

The four-hour RUBY training session was significantly shorter than that described by other breastfeeding peer support programs, many of which offer 20 to 30 hours of training [21]. The content of the training session was similar to that provided to peers in previous successful breastfeeding peer support studies [2]. Based on the success of the RUBY peer support intervention in increasing the proportion of infants receiving breast milk at six months, and the overall positive feedback from peers in terms of their preparation for the role [13], more extensive training is not necessary. However, there may be contextual factors such as background rates of breastfeeding in the community and the peer’s duration of breastfeeding that need to be considered. Data obtained from the Ruby Call Logs does however suggest there is scope for ongoing training to focus on topics raised later in the six-month period of support and the evolving needs of mothers.

Mothers’ information needs evolve over time and this was demonstrated in the data collected in the RUBY Call Logs. Although ‘feed frequency’ remained a consistent topic of conversation throughout the duration of support, ‘nipple pain/ damage’ and ‘positioning and attachment’ were less likely to be raised when the infants were over three months old. In addition, free text responses across all time points indicated that issues related to infant sleep, introducing solids and ‘breastfeeding outside the home’ were raised by mothers. This is consistent with previous studies that have reported how maternal concerns evolve during the early months of breastfeeding. Demirci and Bogen [25] reported positioning and attachment, fatigue, feed frequency and pain were common maternal concerns in the first postpartum week, whereas beyond week six to eight, mothers are more likely to identify perceived milk insufficiency, suspected infant reflux, feed frequency and managing breastfeeding upon return to work as concerns. Concerns regarding milk quantity and infant feeding difficulty including attachment, infant behaviour and nipple refusal may be associated with breastfeeding discontinuation and introduction of formula [26]. In the RUBY study, the mothers receiving peer support reported the most common concerns addressed by their peer supporter were milk supply, normal baby behaviour and effective infant attachment to the breast [27]. The evolution of information needs is not surprising but does highlight that peer training needs to take this into account.

The support provided by peers crosses several domains including appraisal, emotional and informational support [8] . In early work on social support, House identified emotional support as being crucial to conveying the perception of support to others [28]. In the context of breastfeeding, emotional support relates to expression of empathy and connectedness and is not necessarily only related to infant feeding [29]. The lived experience of a phenomenon can be used to create emotional connections and share pragmatic insights, and this has been one of peer support’s strongest mechanisms of action [30]. RUBY volunteers perceived that emotional support was the main reason mothers valued the calls. The mothers in the RUBY study also reported receiving high levels of emotional support [27]. While it is difficult to disentangle and quantify the contribution of informational, emotional and appraisal support, it is important for peers to be aware that guidance and information regarding breastfeeding is only one component of the overall support they will provide.

### Limitations of this study

The data used in this paper were self-reported by RUBY volunteers and Call Log data were limited to those who returned the Call Logs. Personal breastfeeding experiences may have been conflated to ensure acceptance into the program. Volunteers’ perceptions of what the mothers’ main concerns were may not accurately reflect the mother’s intentions. However, the data regarding topics raised by the mother are useful in determining content of training and ensuring the links to additional resource provided in the training manual are relevant.

Topics not included in our pre-coded Call Logs, including ‘infant sleep’, ‘introducing solids’ and ‘breastfeeding outside the home’ could be considered for inclusion in future versions of the Call Log and related resources be made more prominent in the training manual.

The study was undertaken withing the bounds of an RCT in a setting with high breastfeeding initiation. Recruitment may be more challenging outside a research context and in settings with lower breastfeeding rates.

## Conclusions

Given the success of the RUBY intervention in increasing breastfeeding duration in the Australian context, it is important that sufficient details and insights into what was actually delivered are provided, to enable replication of the intervention by those seeking to establish a similar model outside the boundaries of an RCT [5, 31]. This study describes factors related to preparation and support of volunteers in the RUBY RCT that may be relevant to others implementing or scaling up similar interventions. The ABA were an important source of training resources and gave additional credibility to the program. Recruitment via the ABA online platforms generated high levels of interest from potential volunteers. After attending training, most volunteers went on to provide peer support. Peers were supported by a volunteer coordinator.

Future research into the experiences of peers could consider methods that enable exploration of the experiences all peers, throughout the study. In addition to the existing data collected in the Call Log, additional questions exploring the volunteers’ experiences during the period of support may provide more nuanced insights than those collected at the end of the volunteer’s period of participation.

The findings of the RUBY study are important as identifying interventions to increase the duration of breastfeeding has been challenging. The insights shared here will assist those planning breastfeeding peer support training programs and highlights the need for training of peers to meet the evolving information needs of mothers and further reporting of peer breastfeeding characteristics.

## Data Availability

The datasets used and/or analysed during the current study are available from the corresponding author on reasonable request.

## Abbreviations

ABA: Australian Breastfeeding Association
RCT: Randomised controlled trial
